# Computational Study of the Effect of Cortical Porosity on Ultrasound Wave Propagation in Healthy and Osteoporotic Long Bones

**DOI:** 10.3390/ma9030205

**Published:** 2016-03-17

**Authors:** Vassiliki T. Potsika, Konstantinos N. Grivas, Theodoros Gortsas, Gianluca Iori, Vasilios C. Protopappas, Kay Raum, Demosthenes Polyzos, Dimitrios I. Fotiadis

**Affiliations:** 1 Unit of Medical Technology and Intelligent Information Systems, Department of Materials Science and Engineering, University of Ioannina, GR 45110 Ioannina, Greece; vpotsika@cc.uoi.gr (V.T.P.); vprotop@cc.uoi.gr (V.C.P.); 2 Department of Mechanical Engineering and Aeronautics, University of Patras, GR 26500 Patras, Greece; grivas@mech.upatras.gr (K.N.G.); gortsas@mech.upatras.gr (T.G.); polyzos@mech.upatras.gr (D.P.); 3 Berlin-Brandenburg School for Regenerative Therapies, Charité-Universitätsmedizin Berlin, Augustenburger Platz 1, 13353 Berlin, Germany; gianluca.iori@charite.de (G.I.); kay.raum@charite.de (K.R.); 4 Foundation for Research and Technology–Hellas, Institute of Molecular Biology and Biotechnology, Department of Biomedical Research, GR 45110 Ioannina, Greece

**Keywords:** ultrasound, bone modeling, osteoporosis, cortical porosity, basic multicellular units

## Abstract

Computational studies on the evaluation of bone status in cases of pathologies have gained significant interest in recent years. This work presents a parametric and systematic numerical study on ultrasound propagation in cortical bone models to investigate the effect of changes in cortical porosity and the occurrence of large basic multicellular units, simply called non-refilled resorption lacunae (RL), on the velocity of the first arriving signal (FAS). Two-dimensional geometries of cortical bone are established for various microstructural models mimicking normal and pathological tissue states. Emphasis is given on the detection of RL formation which may provoke the thinning of the cortical cortex and the increase of porosity at a later stage of the disease. The central excitation frequencies 0.5 and 1 MHz are examined. The proposed configuration consists of one point source and multiple successive receivers in order to calculate the FAS velocity in small propagation paths (local velocity) and derive a variation profile along the cortical surface. It was shown that: (a) the local FAS velocity can capture porosity changes including the occurrence of RL with different number, size and depth of formation; and (b) the excitation frequency 0.5 MHz is more sensitive for the assessment of cortical microstructure.

## 1. Introduction

Osteoporosis is a bone pathology characterized by low bone mass and structural deterioration of bone tissue leading to bone fragility, thinning of the cortical cortex, reduced bone strength and an increased susceptibility to fractures. Nowadays, the ageing of the population has led to greater prevalence of osteoporosis which is responsible for approximately 2 million fractures which occur annually and may lead to diminished quality of life, disability and even death with a mortality rate 36% depending on the age [1,2]. In clinical practice, the golden standard for the assessment of the disease is dual energy X-ray absorptiometry scan. However, X-ray-based imaging techniques are expensive methods and require radiation exposure. Therefore, the development of non-destructive and non-radiating methods, appropriate to assess the mechanical and structural properties of bone, is crucial.

To this end, several research groups worldwide have investigated the diagnostic and monitoring role of quantitative ultrasound in osteoporotic and fractured bones using experimental, numerical and theoretical means [3,4,5,6,7,8,9]. The research interest in numerical and theoretical methods has allowed the study of phenomena which cannot be observed via experimental measurements extending thus our understanding on the underlying ultrasound propagation mechanisms and the interaction with biological tissues [10]. Specifically, as ultrasound propagates in composite media such as osseous tissues attenuation mechanisms evolve including absorption and scattering phenomena. In soft tissues the viscous forces between neighboring particles moving with different velocities are major sources of acoustic wave absorption [1]. Scattering is induced by the porous nature of cortical bone and the incident energy is partially transferred to the scattered fields. In soft tissues like blood and marrow, ultrasound bulk shear waves are usually ignored as shear waves are highly attenuated at ultrasonic frequencies, while in hard osseous tissues both compression and shear waves are considered.

In addition, the availability of advanced computer-aided computational tools in combination with imaging data derived from micro-computed tomography (μCT) and scanning acoustic microscopy (SAM) have accelerated the evolution of the numerical research [2,3,7,8]. The axial and through transmission techniques have been mainly used to estimate the first arriving signal (FAS) velocity and attenuation as well as to study the propagation of guided waves. In the literature, different thresholds for the detection of the FAS and methodologies to extract guided wave features have been proposed to interpret ultrasound propagation phenomena occurring not only at the periosteal region, but also at deeper bone layers [4,5,6,7,11,12]. More recently, ultrasound reflection and backscattering parameters have been measured to investigate bone microstructure [13,14].

Concerning the first two-dimensional (2D) computational studies, the cortical bone was modeled as a linear elastic homogeneous plate and ultrasound simulations were performed for different plate thicknesses to investigate the thinning of the cortical cortex due to osteoporosis [15,16]. In [17], X-ray computed tomography reconstructions were derived from the human radius in order to develop more realistic 3D computational models of cortical bone. It was shown that for plate thicknesses larger than the wavelength the FAS corresponds to a lateral wave which propagates at the bulk longitudinal velocity of bone, while for very thin plates the FAS wave propagates as the lowest-order symmetric plate mode. More recent 2D and 3D computational studies [2,8,14,17,18,19] take into consideration cortical microstructure, porosity and anisotropy. In [18], SAM images of human femoral neck were used to develop numerical models which account for the sample’s overall shape, microstructure, cortical porosity, heterogeneous matrix elasticity and density. It was found that the FAS velocity is not influenced by trabecular bone properties or by the heterogeneities of the cortical bone mineralized matrix. On the other hand, the FAS was sensitive to variations in cortical porosity. In addition, in [2], SAM human femoral neck cross-sections were used to develop multivariate models for the prediction of pore size, porosity, and cortical thickness. It was shown that the FAS velocity decreases with increasing porosity, while an increase of the cortical thickness and pores’ diameter leads to an increase in FAS velocity. It was also reported that cortical porosity has the strongest effect on the prediction of the FAS velocity. In addition, a 3D micromechanical model consisting of an anisotropic matrix pervaded by cylindrical pores was developed in [19]. It was found that, for the elderly population, the elastic properties of the mineralized matrix do not undergo large variations among different samples, while changes in the intracortical porosity account for most of the variations of mesoscopic elasticity [19].

In this work, we present a 2D computational study of ultrasonic propagation on healthy and osteoporotic cortical bone to examine the effect of porosity on the FAS velocity. We account for the occurrence of large basic multicellular units, simply called non-refilled resorption lacunae (RL), as the predominance of resorption causes an increase in Haversian canal size leading to the increase of cortical porosity and fragility [14,20]. Initially, various microstructural models are established, mimicking normal and pathological tissue states with porosity of 5%, 10% and 16%. Then, numerical models are presented to investigate the impact of changes in the number, size, position and depth of the RL on the FAS. The central excitation frequencies 0.5 and 1 MHz are examined. To our knowledge, this is the first systematic and parametric numerical study which evaluates the FAS velocity variation along cortical bone derived from small, serial propagation paths for different porosities and frequencies, including the occurrence of pores with different sizes.

## 2. Validation of the Numerical Method: A Benchmark Problem

**Figure 1:**
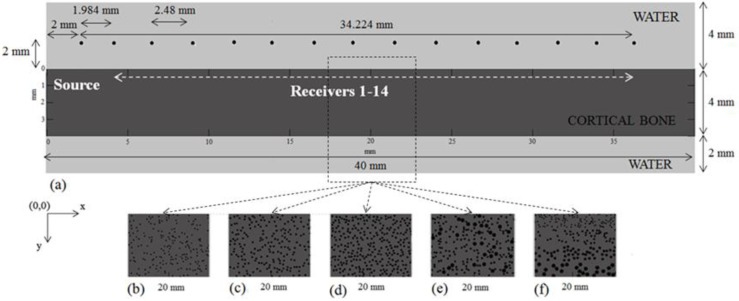
Numerical models of cortical bone corresponding to the first set of simulations (Series I_Po0-16) and Table 2, namely: (**a**) B_Hom; (**b**) B_Po5; (**c**) B_Po10; (**d**) B_Po16; (**e**) B_Po16_RL; and (**f**) B_Po16_Gradual. For each geometry, only one of the three random porosity distributions is depicted in the form of 5 mm cortical segments derived from the original 40mm × 4mm plates. The ultrasound configuration is also presented in (**a**).

**Figure 2:**
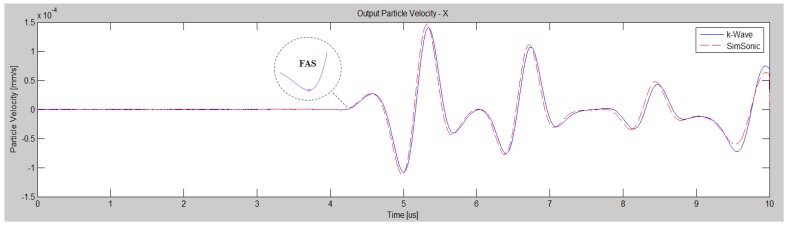
Particle velocity waveforms derived from two different numerical tools, Simsonic and k-Wave, for grid size 20 μm. The threshold for the detection of the FAS is also depicted.

The most popular numerical methods for ultrasonic evaluation of cortical bone are the Finite Difference, Finite Element and Boundary Element methods which solve partial differential equations. According to [21], for time domain modeling of broadband or high-frequency waves these methods can become cumbersome and slow due to the requirements for many grid points per wavelength and small time-steps to minimize unwanted numerical dispersion. In order to validate the finite-difference time-domain (FDTD) code that is used in this work (SimSonic, LIP, Paris, France [22,23]), a benchmark problem was established. We aim to compare the outcome of the FDTD method with a MATLAB (Version, The Mathworks, Inc., Natick, MA, USA) toolbox which combines pseudo-spectral and k-space methods, so the spatial gradients are calculated by using Fast Fourier Transforms rather than by using a finite difference stencil (k-Wave, [21]). The computational model of Figure 1a was used corresponding to an intact cortical plate with thickness of 4 mm and length 40 mm surrounded by water. The material properties of bone and water are presented in Table 1. The grid size was set to 20 μm and the wave propagation simulation time was 10 μs. A point source and receiver were used to calculate the velocity at a distance of 12.5 mm between the transducers. The transducers were placed directly onto the cortical surface. A 1-MHz Gaussian pulse was used as the excitation signal. According to the results of Figure 2, the two waveforms have the same time of arrival of the FAS as well as the same qualititative behavior over time enhancing the accuracy of the FDTD method.

In order to provide quantitative information for the differences of the two waveforms, a convergence study is conducted for the grid sizes of 50, 30 and 20 μm. The methodology of waveform rectification was used followed by numerical integration via the trapezoidal method. A full-wave rectifier converts the examined waveform to a waveform of constant polarity (positive or negative). Specifically, the excitation signal was rectified as well as the received waveforms from k-Wave and Simsonic. Numerical integration was applied using the trapezoidal method to derive the emitted and received signal area. Next, we calculated the ratio of the emitted signal area to the received waveforms’ area, which is a quantitative indicator reflecting the whole area of interest. If we denote the calculated ratio with *E_sim_* for Simsonic and *E_kw_* for k-Wave, the corresponding values were: (a) *E_kw_* = 1166, *E_sim_* = 1084 (difference_50μm_ = 82) for 50 μm, (b) *E_kw_* = 1935, *E_sim_* = 1921 (difference_30μm_=14) for 30 μm and (c) *E_kw_* = 2924, *E_sim_* = 2919 (difference_20μm_ = 5) for 20 μm.

## 3. Numerical Evaluation of Cortical Porosity Using Ultrasonic Techniques

### 3.1. Model Geometry

In this section, we present the structural features of the computational models representing different cases of cortical porosity and pores’ sizes as well as different number and distributions of RL. The structural and material properties were derived from [14], in which the Haversian canals of normal size were differentiated from the RLs to distinguish healthy bones (with no or few RL) from degenerated ones. As can be seen in Figure 1, the cortical bone was modeled as a 2D plate (length 40 mm, width 4 mm) surrounded by water (upper surface 4 mm, lower surface 2 mm).

Two sets of numerical simulations were performed in order to evaluate: (a) porosity changes from 0% to 16% covering the whole region of cortical bone (Series I_Po0-16), (b) the occurrence of a single or a cluster of RL in small propagation paths (Series II_RL). The selection of the porosities of Series I_Po0-16, and the sizes of the RL were based on previous studies investigating the interaction of ultrasound with cortical porosity [2,5,10,14]. However, it should be emphasized that the evaluation of cortical porosity depends on several parameters such as age, gender and region of interest [5]. The first set is presented in Figure 1, in which cortical bone was modeled both as a homogeneous and nonhomogeneous medium to account for the cases of: (a) intact bone (Figure 1a); (b) porosity 5% (Figure 1b); (c) porosity 10% (Figure 1c); and (d) porosity 16% (Figure 1d–f). In Table 2, we present the exact structural features concerning the porosity, number and dimensions of the normal and larger pores. For each microstructural scenario of Table 2, a total of three numerical models were established and the mean local FAS velocity values were calculated as well as the standard error.

Circular scatterers were considered in the transverse direction and the selected radii of normal pores and RL are presented in Table 2. Given the porosity and the dimensions of the plate and scatterers, the number of the pores can be calculated. An algorithm was developed to generate the osseous and soft tissues as greyscale values and the “porosity maps” with a random distribution of the pores. The scatterers were not in contact and did not merge with each other as well as with the cortical boundaries.

**Figure 3:**
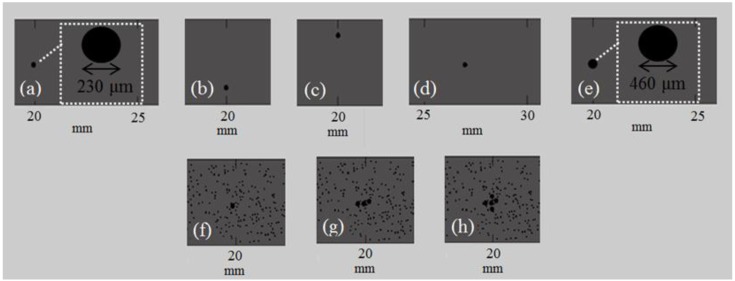
Numerical models of cortical bone corresponding to the second set of simulations (Series II_RL) focusing on the regions of the occurrence of RL: (**a**) homogeneous bone, 1 RL with center coordinates (*x*, *y*) = (20, 2); (**b**) homogeneous bone, 1 RL with center coordinates (*x*, *y*) = (20, 3); (**c**) homogeneous bone, 1 RL with center coordinates (*x*, *y*) = (20, 1); (**d**) homogeneous bone, 1 RL with center coordinates (*x*, *y*) = (27, 2) mm; (**e**) homogeneous bone, 1 RL with center coordinates (*x*, *y*) = (20, 2) and double the diameter; (**f**) B_Po5_RL1; (**g**) B_Po5_RL3; (**h**) B_Po5_RL5.

Figure 3 illustrates cortical segments of Series II_RL in which the cortex of a long bone was modeled initially as a 2D homogeneous plate including the presence of 1 RL at different lengths, depths and diameters (Figure 3a–e). Then, in Figure 3f–h, we account for cortical microstructure to simulate the case of normal remodeling (Table 2: cases B_Po5_RL1, B_Po5_RL3, B_Po5_RL5) including a single or a cluster of RL in the center of the plate.

### 3.2. Material Properties

The cortical bone was modeled as an isotropic, linear elastic medium. We assumed that the soft tissues surrounding the cortical plate as well as the circular pores are composed of water. Table 1 summarizes the material properties assigned to the cortical bone, scatterers and soft tissues which were derived from [14].

### 3.3. Ultrasound Configuration

Simulations of ultrasound propagation were performed in the tangential direction (Figure 1a). Calculations were conducted by placing one point source and 14 point receivers: (a) at a distance 2 mm from the upper cortical layer (non-contact transducers); (b) directly onto the cortical cortex (implanted transducers). The distance between two successive receivers was 2.48 mm implying that the distance between the emitter and the receivers ranges from 1.984 to 34.224 mm (receiver 1 (R1) – receiver 14 (R14)). The width of each transducer was equal to 11 elements. The use of a linear array of transducers is in agreement with exsisting ultrasonic devices for bone characterization such as the bidirectional device in [24].

### 3.4. Determination of the Ultrasonic Wave Propagation Path and Velocity

The “local FAS velocities” correspond to small propagation paths and were calculated as the difference in the FAS arrival time of the signals from successive receivers (distance 2.48 mm). In this way, we intend to estimate the structural changes along small regions of cortical bone due to porosity variations or to the presence of larger pores. When multiple receivers are considered, the change in the FAS arrival time *Δt* (μs) can be calculated using *Δt(x)* = *t(x_i_)* − *t(x_i-1_)*, where i is the number of the receiving position, *t(x_i_)* is the arrival time of the signal at receiver i and *t(x_i-1_*) is the arrival time at the previous receiver [25]. For the detection of the FAS a threshold was applied to the receiving waveforms corresponding to the identification of the first signal extremum (Figure 2) [26].

### 3.5. Boundary Conditions

The cortical cortex was surrounded by soft tissues and perfectly matched layers (PML) were used to limit spurious reflections from the boundaries simulating an infinitely long model. The PML efficiency was set to 80 dB which means that the wave reflected by the PML is expected to be 80 dB below the amplitude of a incident wave, in the case of normal incidence [22,23].

### 3.6. Numerical Simulation and Signal Analysis in the Time Domain

The numerical solution of the 2D wave propagation problem was carried out by using an FDTD code [22,23]. The computations are based on a system of elastodynamic equations corresponding to the propagation of mechanical waves in continuous media which obey Hooke's law and are expressed as:

(1) $$\rho \left(x\right)\frac{\partial u_{i}}{\partial t}\left(x,t\right)=\overset{d}{\underset{j=1}{\sum }}\frac{\partial T_{ij}}{\partial x_{j}}\left(x,t\right)+f_{i}\left(x,t\right)$$

(2) $$\frac{\partial T_{ij}}{\partial t}\left(x,t\right)=\overset{d}{\underset{k=1}{\sum }}\overset{d}{\underset{l=1}{\sum }}c_{ijkl}\left(x\right)\frac{\partial u_{k}}{\partial x_{l}}\left(x,t\right)+\theta_{ij}\left(x,t\right)$$

where subscripts i={1,…,d} refer to the direction of space and d to the space dimension (*d* =2 for 2D computational models), x and t are the space and time variables, *ρ(x)* is the mass density and c(x) is the fourth-order rigidity tensor. These parameters entirely define the material properties and geometry of the medium. *u_i_* (*x*, *t*) are the vector components of the particle velocity field, *T_ij_* (*x*, *t*) are the components of the stress tensor, while f_i_ denote the vector components of force sources and *θ_ij_* denote the tensor components of strain rate sources.

A Hanning pulse was used as the excitation signal including four sinusoidal cycles [27]. The examined central angular frequencies were 0.5 and 1 MHz. The duration of the simulations was 25 μs. The accuracy of the solution depends on the relation between the element size and the wavelength. According to the software requirements, the stability condition is defined as:

(3) $$\Delta t\leq \frac{1}{\sqrt{d}}\frac{\Delta x}{c_{max}}$$

where *Δt* and *Δx* denote the time and spatial steps used to approximate time or spatial derivatives, c_max_ is the largest speed of sound amongst all the simulated materials (*c_max_* = *c_bone_* = 3939 m/s), and *d* refers to the space dimension (*d* = 2). The grid size was set to 8 μm, and a convergence study is presented in the following subsection to demonstrate the accuracy of the findings.

#### Convergence Study

The grid size must be dense enough to ensure an accurate numerical solution and to minimize the computational cost. In addition, it must be small enough in comparison to the diameter of the pores. The convergence study is based on the numerical model B_Po5 which integrates the smallest scatterers among all the geometries of Table 2. Two transducers were considered which were placed directly onto the cortical surface and their distance was set to 20 mm. FDTD simulations were conducted and the FAS velocities were calculated for the central excitation frequency 1 MHz and the grid step sizes 4, 8, and 16 µm. The calculated FAS values were 3499 m/s for 4 μm, 3495 m/s for 8 μm, and 3489 m/s for 16 μm. It can be concluded that the 8-μm grid size is a good compromise for an accurate numerical solution and less time consuming simulations which is in agreement with [2].

### 3.7. Statistical Analysis

A statistical analysis was conducted and the results are presented as local mean FAS velocity ± standard error. Linear regression analysis and one-way analysis of variance (one-way ANOVA) were used to evaluate changes in porosity. The statistical findings were considered significant for *p*-values less than 0.05 [9].

## 4. Results

**Figure 4:**
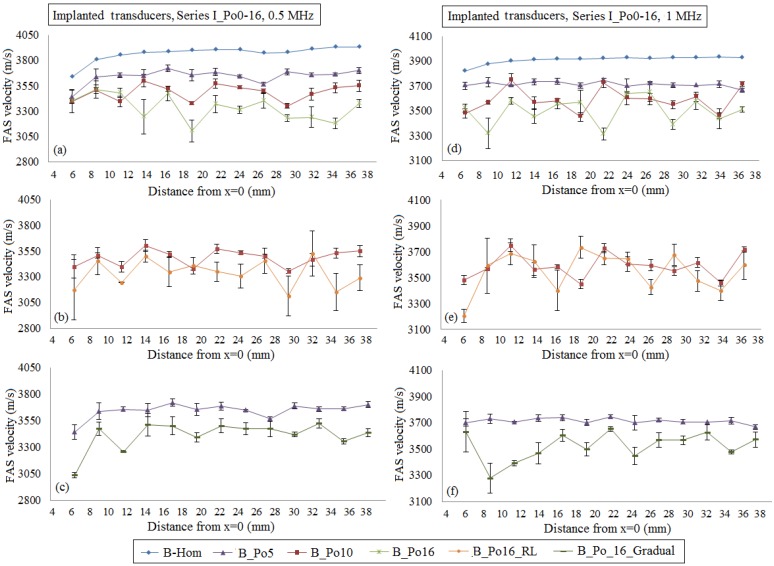
The potential of the mean local FAS velocity to detect changes in cortical porosity from 0%–16%. The diagrams correspond to the first set of simulations under the assumption of implanted transducers and the excitation frequencies: (**a**)–(**c**) 0.5 MHz; and (**d**)–(**f**) 1 MHz. The standard error bars demonstrate the FAS velocity variation for the three numerical models established for each porosity scenario.

**Figure 5:**
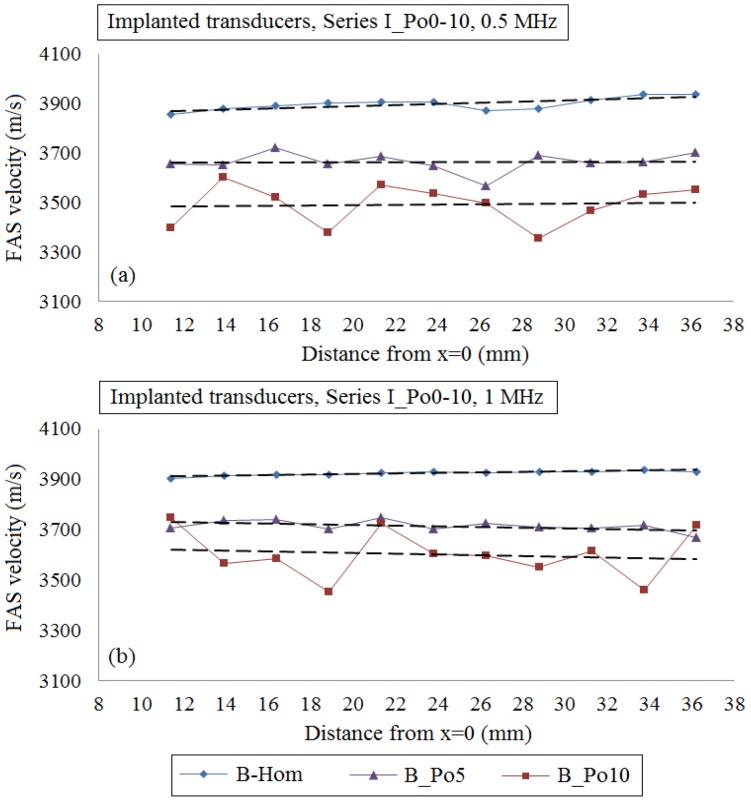
Linear fit derived from multiple receiving positions for cortical porosities from 0 to 10%. The diagrams correspond to the first set of simulations under the assumption of implanted transducers and the excitation frequencies: (**a**) 0.5 MHz; and (**b**) 1 MHz. The corresponding equations, coefficients of determination and root mean square errors are presented in Table 3 and Table 4.

**Figure 6:**
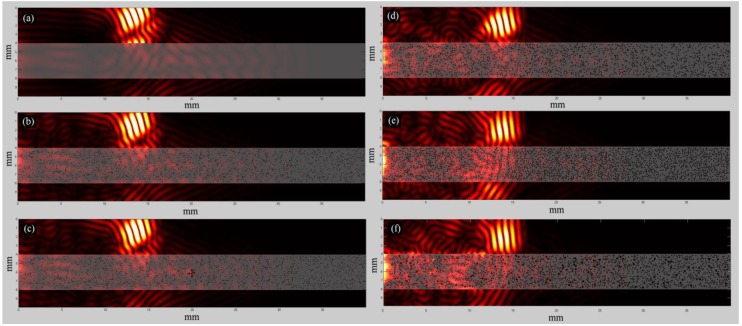
Snapshots of wave propagation for the first set of simulations using non-contact transducers for the excitation frequency of 1 MHz and time instant 10 μs for the cases: (**a**) B_Hom; (**b**) B_Po5; (**c**) B_Po5_RL5; (**d**) B_Po10; (**e**) B_Po16; (**f**) B_Po16_RL.

**Figure 7:**
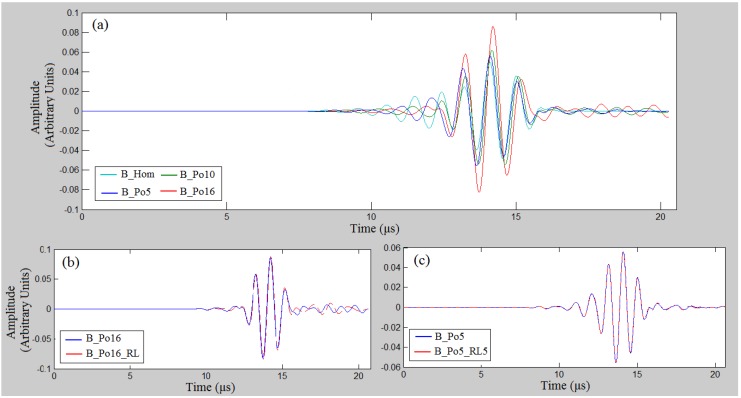
Waveforms derived from receiver R8 for the excitation frequency 1 MHz and simulation time 20 μscomparing the examined cases: (**a**)B_Hom, B_Po5, B_Po10 and B_Po16; (**b**) B_Po16 and B_Po16_RL; (**c**) B_Po5 and B_Po5_RL5.

**Figure 8:**
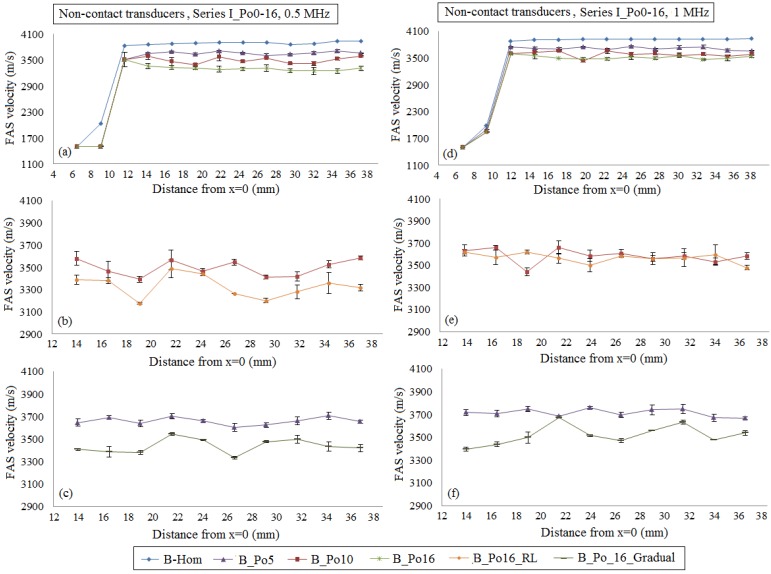
The potential of the mean local FAS velocity to detect changes in cortical porosity from 0% to 16%. The diagrams correspond to the first set of simulations when the transducers are placed at a distance of 2 mm from the cortical cortex. The results for the excitation frequencies : (**a**)–(**c**) 0.5 MHz; and (**d**)–(**f**) 1 MHz are presented. The standard error bars demonstrate the FAS velocity variation for the three numerical models established for each porosity scenario.

**Figure 9:**
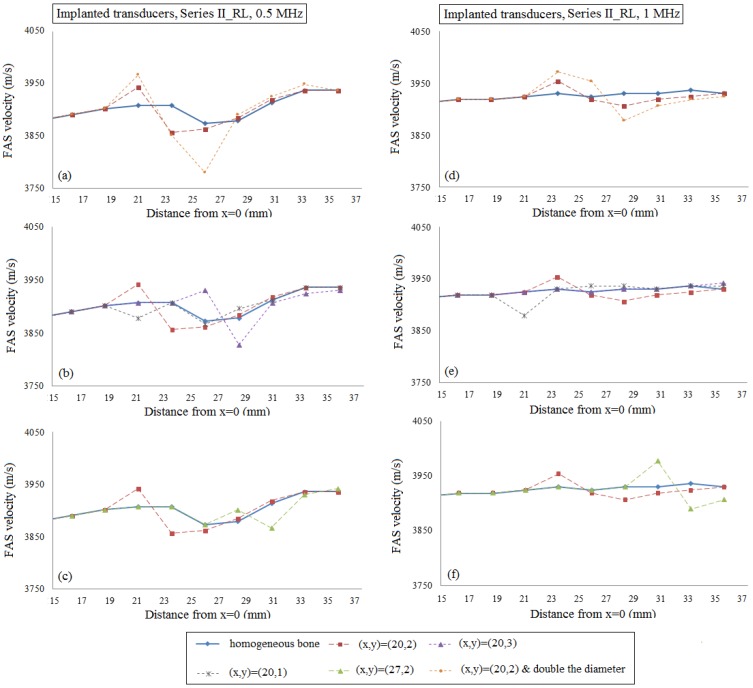
The potential of the local FAS velocity to detect the occurrence of a single RL considering cortical bone as a homogeneous medium (geometries illustrated in Figure 3a–e). The diagrams correspond to the second set of simulations under the assumption of implanted transducers and the excitation frequencies: (**a**)–(**c**) 0.5 MHz; and (**d**)–(**f**) 1 MHz. The legend describes the center coordinates of the RL.

**Figure 10:**
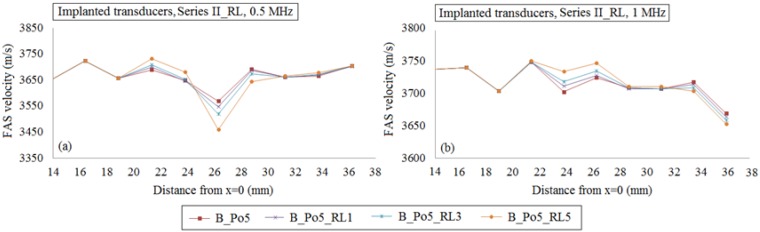
The potential of the local FAS velocity to detect the occurrence of a single or a cluster of RL considering cortical bone as a nonhomogeneous medium for the cases B_Po5, B_Po5_RL1, B_Po5_RL3 and B_Po5_RL5 (geometries illustrated in Figure 3f–h). The diagrams correspond to the second set of simulations under the assumption of implanted transducers and the excitation frequencies: (**a**) 0.5 MHz; and (**b**) 1 MHz.

**Figure 11:**
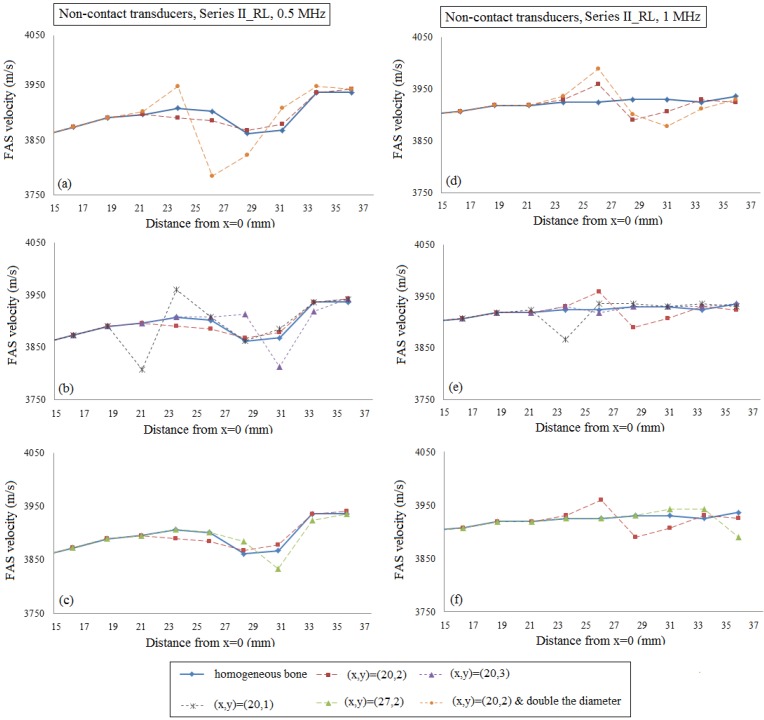
The potential of the local FAS velocity to detect the occurrence of a single RL considering cortical bone as a homogeneous medium (geometries illustrated in Figure 3a–e). The diagrams correspond to the second set of simulations when the transducers are placed at a distance of 2 mm from the cortical cortex. The results for the excitation frequencies : (**a**)–(**c**) 0.5 MHz; and (**d**)–(**f**) 1 MHz are presented. The legend describes the center coordinates of the RL.

**Figure 12:**
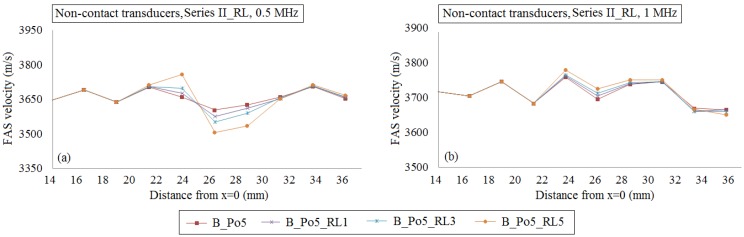
The potential of the local FAS velocity to detect the occurrence of a single or a cluster of RL considering cortical bone as a nonhomogeneous medium for the cases B_Po5, B_Po5_RL1, B_Po5_RL3 and B_Po5_RL5 (geometries illustrated in Figure 3f–h). The diagrams correspond to the second set of simulations when the transducers are placed at a distance of 2 mm from the cortical cortex. The excitation frequencies: (**a**) 0.5 MHz; and (**b**) 1 MHz are examined.

Figure 4, Figure 5, Figure 6, Figure 7, Figure 8, Figure 9, Figure 10, Figure 11 and Figure 12 present the results derived from the two simulation sets of Figure 1 and Figure 3 and the central excitation frequencies 0.5 and 1 MHz. As multiple receivers were considered and small propagation paths were examined, the horizontal axis represents the distance of each receiving element from the starting point of the plate with coordinates (*x*, *y*) = (0, 0). First, in Subsection 4.1, we present the results derived for porosity changes from 0% to 16%, and then, in Subsection 4.2, the findings corresponding to the occurrence of a single or a cluster of RLs in small propagation paths at different depths, lengths and sizes.

### 4.1. First Set of Simulations (Series I_Po0-16)

#### 4.1.1. Implanted Transducers

Figure 4 presents the FAS velocity variation profile from multiple implanted receivers if we consider cortical bone as a nonhomogeneous medium with porosity from 0 to 16%. The local FAS velocities were calculated as the mean values from the same receiving position of three simulation maps with different random distributions of the pores keeping the same microstructure characteristics. The mean FAS velocities and the standard error bars for the excitation frequency 0.5 MHz are presented in Figure 4a–c and for 1 MHz in Figure 4d–f. It can be observed that a porosity increase leads to the decrease of the FAS velocity variation profile, and this is more apparent for the excitation frequency 0.5 MHz.

More specifically, if we consider the case B_Hom as the reference case, the relative percentage differences in Figure 4a for the cases B_Po5, B_Po10 and B_Po16 were calculated in the range 4.32%–7.87%, 6.58%–13.53% and 6.41%–20.34%, respectively. These findings can be directly compared with Figure 4d. For the excitation frequency 1 MHz, the relative percentage differences for the cases B_Po5, B_Po10 and B_Po16 were from 3.14% to 6.67%, 3.91%–12.10% and 7.35%–15.56%, respectively. Then, Figure 4b,c,e,f present more realistic scenarios of pathology including both normal pores and RLs for the case B_Po16_RL (Figure 4b for 0.5 MHz and Figure 4e for 1 MHz) and the gradual formation of osteoporosis (Figure 4c for 0.5 MHz and Figure 4f for 1 MHz). In Figure 4b, a porosity increase up to 16% including the occurrence of RLs (B_Po16_RL) leads to a decrease of the local FAS velocities and the relative percentage differences were from 9.23% to 19.71% for the excitation frequency 0.5 MHz. In addition, we observe that there are few positions (e.g. Figure 4b, *x* = 30–32 mm) which show a minor increase of the FAS velocity locally despite the porosity increase. Figure 4e presents the FAS velocities for B_Po16_RL and the excitation frequency 1 MHz. It can be seen that as the frequency increases it is more difficult to discriminate a specific FAS velocity behavior among the cases B_Po10 and B_Po16_RL. Finally, Figure 4c,f show the potential of the local FAS velocity to detect the gradual formation of osteoporosis. The diagram B_Po5 is depicted in the same diagram with B_Po16_Gradual as when osteoporosis is gradually formed the upper cortical layer has the microstructure of a healthy bone, while the porosity and pores’ size increase starts from the endosteal region. The relative percentage differences for the case B_Po16_Gradual keeping B_Hom as the reference were calculated from 8.70% to 16.63% for the excitation frequency 0.5 MHz (Figure 4c) and from 5.02% to 15.49% for 1 MHz (Figure 4f), respectively.

Then, one-way ANOVA was conducted and the *p*-value was calculated. It was found to be lower than 0.05 (*p* ≈ 0), implying that there are significant differences between group means. Linear regression analysis was also performed, as in some receiving positions of Figure 4 the mean FAS velocity was found to increase locally despite the porosity increase. Figure 5 shows that linear regression analysis is not influenced by local phenomena, and the examined cases can be discriminated. The corresponding equations, coefficients of determination (R^2^) and root mean square errors (RMSE) are included in Table 3 and Table 4. All the results are not presented graphically to reduce complexity, as similar findings to Figure 5 were derived.

#### 4.1.2. Transducers at a Distance of Two Millimeters from the Cortical Cortex

Figure 6 presents snapshots of ultrasound wave propagation for indicative cases considering the excitation frequency 1 MHz and time instant 10 μs. It is shown that, as porosity increases, multiple scattering mechanisms evolve (Figure 6d,e,f). In addition, it can be observed that the application of PML limits spurious reflections from the boundaries, but the phenomenon is not totally diminished.

Then, in Figure 7 the received waveforms are illustrated derived from receiver R8, which is placed above the region of the cluster of 5 RLs (Figure 3h) for indicative cases and the excitation frequency 1 MHz, It can be seen in Figure 7a that the increase of porosity induces a delay in the propagation of the signal if we compare the zero-crossing times. An increase in amplitude was also found with increasing the porosity. A comparison of the waveforms of the cases B_Po16 and B_Po16_RL is presented in Figure 7b, showing that the occurrence of the larger pores has an effect on the received waveforms. However, in Figure 7c, the waveforms for the cases B_Po5 and B_Po5_RL5 almost coincide, while no difference can be observed at the corresponding snapshots of Figure 6.

Figure 8 presents the mean FAS velocities and standard error bars when the transducers are placed at a distance of 2 mm from the cortical surface. The porosities from 0% to 16% are examined as in Figure 4. In Figure 8a,d, low FAS velocities are observed for the receivers which are placed before *x* = 12 mm due to the direct propagation of the FAS via the soft tissues.

More specifically, if we consider the case B_Hom as the reference case, the relative percentage differences in Figure 8a for 0.5 MHz and B_Po5, B_Po10 and B_Po16 were calculated in the range 4.71%–7.65%, 7.22%–12.83% and 12.90%–17.60%,respectively. For the excitation frequency 1 MHz (Figure 8d), the relative percentage differences for B_Po5, B_Po10 and B_Po16 were from 4.24% to 6.89%, 6.37%–12.19% and 8.78%–11.82%, respectively. Then, Figure 8b,e corresponds to the case B_Po16_RL and are directly compared with the case B_Po10. Keeping B_Hom as the reference case the relative percentage differences for B_Po16_RL were from 10.51 to 18.33% for 0.5 MHz (Figure 8b) and 7.17%–11.62% for 1 MHz (Figure 8e). Thus, even if according to Figure 7b, the two waveforms seem to coincide for propagation time less then 10 μs, however the calculation of the FAS in small propagation paths shows quantitative differences when large pores are considered. The results for the gradual distribution of the pores are shown in Figure 8c,f. The relative percentage differences for B_Po16_Gradual were derived from 9.02% to 14.55% for the excitation frequency 0.5 MHz (Figure 8c) and from 6.31% to 12.95% for 1 MHz (Figure 8f), respectively. It should be mentioned that in Figure 8b,c,e,f, we do not present the whole propagation path, as the initial low values of Figure 8a,b corresponding to the direct FAS propagation through the water were neglected. In addition, linear regression analysis was performed and the results are depicted in Table 3 and Table 4. It was shown that the examined cases can be discriminated even for the diagrams of Figure 8e in which the mean local FAS velocities do not show a specific behavior with increasing the porosity.

Concerning one-way ANOVA, the *p*-value was lower than 0.05 (*p* ≈ 0) ignoring the receiving positions near the source (*d* < 12 mm). The reason for not considering the first receivers in the calculations is that as it can be seen in Figure 8a,d for *d* < 12 mm, the FAS wave propagates directly in the soft tissues and low velocities are derived in comparison to the bulk longitudinal velocity of bone. This assumption was also considered for the cases of Figure 4 to keep the same conditions.

### 4.2. Second Set of Simulations (Series II_RL)

#### 4.2.1. Implanted Transducers

This section presents the results corresponding to the occurrence of: (a) a single RL at different depths, lengths and sizes under the assumption of cortical homogeneous bone (Figure 9 and Figure 11); (b) a single or a cluster of RL taking into account cortical microstructure (Figure 10 and Figure 12). In Figure 9 and Figure 10, implanted transducers are used and the excitation frequencies 0.5 and 1 MHz are examined. Figure 9a–c depict the local FAS velocity variation profile for the excitation frequency 0.5 MHz and the presence of a single RL. For comparison purposes, all the diagrams include the calculated values for the intact bone (blue line) and the case of a single RL with center coordinates (*x*, *y*) = (20, 2) (red line). All the diagrams start from a distance of 15 mm in the *x* axis as for lower cortical lengths all the curves coincide. In Figure 9a, we observe that for 0.5 MHz the occurrence of one RL in the center of the plate can be captured by the receivers which are placed directly above the region of interest showing an increase of the FAS velocity of 35 m/s compared to the homogeneous bone diagram. In addition, if we keep the same RL position and double the diameter of the pore (orange line), the receivers which are placed in the position *x* = 19–21 mm are the first to detect an increase of the FAS velocity of 58 m/s in comparison to the homogeneous bone value. In Figure 9b, we examine how the depth of the RL occurrence influences the FAS velocity. It can be seen that, if we place the RL at a higher cortical surface (grey line), the receivers which first capture the presence of a pore are placed again in the propagation path between *x* = 19–21 mm showing a velocity decrease of 29 m/s comparing to the reference case of homogeneous bone. On the other hand, if we increase the depth from the cortical surface (purple line), there is a delay in the identification of the RL by the receivers above the region of *x* = 25–21 mm showing an increase of the FAS velocity of 57 m/s. Finally, Figure 9c shows that if we change the RL position along the *x* axis, the receivers which are placed above the region of interest are the first to detect an increase of the FAS velocity of 23 m/s revealing the position of the RL formation.

Figure 9d–f presents the local FAS velocity variation profile for the excitation frequency 1 MHz and the presence of a single RL. In Figure 9d, it can be seen that the occurrence of a single RL in the center of the plate (red line) can be identified with a delay by the receivers which follow the region of interest (around *x* = 23 mm) showing an increase of the FAS velocity of 23 m/s. In addition, it was found that if we double the diameter of the RL (orange line), the same receivers are the first to detect an increase of the FAS velocity of 41 m/s. Then, Figure 9e indicates that by increasing the depth from the cortical surface (purple line), the larger pore cannot be detected as this diagram coincides with the case of homogeneous bone (blue line). On the other hand, the occurrence of the RL near the upper cortical surface (grey line) leads to a decrease of the FAS velocity of 46 m/s derived from the receivers directly above the region of interest. Finally, Figure 9f shows that if we change the position of the RL along the *x* axis, the receivers which are placed approximately at a distance of 3 mm after the pore are the first to capture an increase of the FAS velocity of 47 m/s.

Then, in Figure 10, we investigate the potential of the local FAS velocity to detect the occurrence of a single or a cluster of three or five large pores in the center of the plate taking into account the nonhomogeneous nature of cortical bone. Figure 10a presents the FAS velocity variation profile for the excitation frequency of 0.5 MHz, while Figure 10b the FAS values for 1 MHz considering the diagram of B_Po5 (red line) as the reference case. For the excitation frequency of 0.5 MHz the relative percentage differences were calculated in the range: (a) 0.04%–0.28% for B_Po5_RL1, (b) 0.04%–1.36% for B_Po5_RL3 and (c) 0.04%–3.08% for B_Po5_RL5. It can be seen that the first receivers which can identify the RL occurrence are placed above the region of interest (*x* = 20–22 mm). A 2-mm delay in the detection of the larger pores is observed in Figure 10b for 1 MHz as they are first identified from the receivers in the region *x* = 22–24 mm. Lower relative percentage differences were calculated compared to 0.5 MHz from: (a) 0.05%–0.24% for B_Po5_RL1, (b), 0.23%–0.42% for B_Po5_RL3, and (c) 0.05%–0.85% for B_Po5_RL5.

#### 4.2.2. Transducers at a Distance of Two Millimeters from the Cortical Cortex

Figure 11a–c present the local FAS velocity variation profile for the excitation frequency 0.5 MHz and the presence of a single RL by placing the transducers at a distance of 2 mm from the cortical cortex. All the diagrams include the case of intact homogeneous bone (blue line) and the case of a single RL with center coordinates (*x*, *y*) = (20, 2) (red line). In Figure 11a, it can be seen that, for 0.5 MHz, the occurrence of one RL in the center of the plate can be captured by the receivers which are placed directly after the region of interest (between *x* = 21–23 mm) showing a decrease of the FAS velocity of 17 m/s compared to the homogeneous bone diagram. Then, if we keep the same RL position and double the diameter of the RL (orange line) the same receivers are the first to detect an increase of the FAS velocity of 41 m/s in comparison to the homogeneous bone value. Figure 11b shows the impact of the RL depth on the FAS values. We observe that if we place the RL at a higher cortical layer (grey line), the receivers which first capture the presence of a pore are placed directly above the region of interest showing a velocity decrease of 89 m/s comparing to the reference case of homogeneous bone. However, if we increase the depth from the cortical cortex (purple line), the receivers which are placed at a distance approximately 5 mm from the RL are the first to identify its occurrence showing an increase of the FAS velocity of 51 m/s. Finally, Figure 11c shows that if we change the RL position along the *x*-axis, the receivers which are placed above the region of interest are the first to capture an increase of the FAS velocity of 23 m/s revealing the position of the larger pore.

Additionally, Figure 11d–f present the local FAS velocity variation profile for the excitation frequency 1 MHz and the presence of a single RL. Specifically, Figure 11d shows that the occurrence of a single RL in the center of the plate (red line) can be identified with a delay by the receivers which follow the region of interest (around *x* = 23–25 mm) showing an increase of the FAS velocity of 35 m/s. In addition, it can be observed that if we double the diameter of the RL (orange line) the same receivers are the first to identify an increase of the FAS velocity of 65 m/s. In Figure 11e, we observe that if we increase the depth from the cortical surface (purple line) the larger pore cannot be detected as this diagram almost coincides with the case of homogeneous bone. On the contrary, the occurrence of the RL near the upper cortical surface (grey line) leads to a decrease of the FAS velocity of 57 m/s, and it can be identified with a small delay by the receivers placed above the propagation path between *x* = 21–23 mm. Finally, Figure 11f shows that, if we change the position of the RL along the *x*-axis, the receivers which are placed between *x* = 29–31 mm are the first to detect an increase of the FAS velocity of 17 m/s.

Then, in Figure 12, we investigate the potential of the local FAS velocity variation profile to identify the occurrence of a single or a cluster of three or five large pores in the center of the plate taking into account the cortical microstructure. Figure 12a illustrates the local FAS velocities for the excitation frequency 0.5 MHz, while Figure 12b shows the FAS values for 1 MHz considering B_Po5 (red line) as the reference case. For the excitation frequency 0.5 MHz, the relative percentage differences were calculated in the range: (a) 0.05%–0.75% for B_Po5_RL1, (b) 0.05%–1.41% for B_Po5_RL3 and (c) 0.18%–2.71% for B_Po5_RL5. It can be observed that the first receivers which capture the RL are placed between *x* = 21–23 mm showing a short delay. Α 4-mm delay in the detection of the larger pores can be seen in Figure 10b for 1 MHz and lower relative percentage differences were calculated compared to 0.5 MHz from: (a) 0.04–0.32% for B_Po5_RL1, (b) 0.14–0.46% for B_Po5_RL3, (c) 0.05%–0.79% for B_Po5_RL5, respectively.

## 5. Discussion

This work presents a 2D parametric and systematic computational study aiming to investigate the effect of cortical porosity on ultrasound wave propagation in healthy and osteoporotic long bones. In comparison to previous studies [2,14,26], we established more realistic scenarios, as this is the first time that the distribution of the pores was randomized including normal pores, as well as pores with diameters larger than the Haversian canal size. Moreover, the analysis was not limited to a small segment of cortical bone with the presence of only one large basic multicellular unit as in [14]. The gradual formation of osteoporosis was also considered by simulating more physiological distributions of pores with larger pores in the endosteal cortex and smaller ones in the periosteum. The excitation frequencies 0.5 MHz and 1 MHz were examined to investigate which frequency is more sensitive to detect changes in cortical porosity and the occurrence of RLs. Numerical simulations of wave propagation were performed in the tangential direction and various microstructural models mimicking normal and pathological tissue states were investigated. In comparison to traditional FAS velocity measurements using one source and one receiver, we performed calculations of “local” FAS values in small successive propagation paths along the cortical cortex in order to establish possible guidelines for the interaction of ultrasound with cortical microstructure.

Initially, in Figure 4a,d, and Figure 8a,d, we examined the FAS velocity variation profile along cortical bone for microstructural models with porosities from 0 to 16%. It was made clear that as the porosity increases, the FAS velocities calculated in small propagation paths decrease for both the examined frequencies. However, the relative percentage differences calculated considering B_Hom as the reference case were higher for 0.5 MHz comparing to 1 MHz. Moreover, Figure 7a shows a delay in the time of arrival with increasing the porosity followed by an amplitude increase. This may be explained by the reflection mechanisms, which enhance the received signal for higher porosities. However, despite the significance of the calculation of the FAS velocities from the first signal extremum, the evaluation of the whole waveform would be a possible direction for future research based on guided wave analysis or on the waveform rectification followed by numerical integration via the trapezoidal method (presented in Section 2).

Additionally, the use of implanted transducers led to higher relative percentage differences compared to the use of non-contact transducers, while a delay in the detection of a single or a cluster of RL was observed for non-contact transducers. This can be attributed to the attenuation mechanisms induced by the presence of the soft tissues in the propagation path. The attenuation mechanisms include the evolution of absorption and scattering phenomena. Scattering is induced in bone due to the presence of the pores while soft tissues are major sources of acoustic wave absorption. Despite the accuracy of the FDTD method, which was demonstrated via a convergence study, a limitation of the present work is the simulation weakness of the software to account for attenuation effects resulting from the viscoelasticity [2]. In addition, it should be mentioned that the use of implanted transducers is considered as an invasive procedure for osteoporosis and was examined only for comparison purposes with the non-contact transducers. In clinical practice, their insertion is easy for fracture healing assessment [12,27], but, for the evaluation of osteoporosis, this configuration is not applied during *in vivo* measurements.

Concerning the case of pathology with porosity of 16%, two additional scenarios were investigated to account for the presence of pores with different diameters as well as for the gradual formation of osteoporosis. It was found that the FAS velocity variation profile shows a decrease along the cortical cortex for both B_Po16_RL and B_Po16_Gradual for 0.5 MHz. On the other hand, the excitation frequency of 1 MHz was not convenient for B_Po16_RL (Figure 4e and Figure 8e) as no specific FAS velocity tendency was observed, while, for B_Po16_Gradual, it was sensitive to the gradual porosity increase. A direct comparison of the received signals from B_Po16 and B_Po16_RL was also included in Figure 7b showing a difference in the received signals due to the occurence of large pores.

Additionally, a statistical analysis was conducted based on the estimation of the standard errors, one-way ANOVA and linear regression analysis. It should be mentioned that the receiving positions near the source (*d* < 12 mm) were ignored as in Figure 8a,d, we observe that for short distances between the transducers, low velocities are calculated, revealing that the FAS propagates directly via water. The calculated *p*-values were lower than 0.05 indicating that there are statistically significant differences between group means as the porosity increases from 0% to 16%. The low *p*-values can be explained by the high dispersion of the FAS velocity derived from serial measurements for porosity of 16%. Linear regressions analysis revealed a specific FAS velocity trend even for the cases of Figure 4e and Figure 8e. Nevertheless, low R^2^ values were derived implying that this analysis could be used only for supplementary observations following traditional local and successive FAS velocity measurements. The establishment of three numerical models for each case is another parameter which requires further discussion. The use of three models is considered as a good compromise to reduce the computational cost and derive accurate conclusions taking into account the calculated standard error values. More specifically, for all the examined cases after three runs, the maximum FAS velocity variation was far less than 5%, thus we decided not to make more runs to keep calculations and simulations simpler.

Then, the case of normal remodeling was examined including the presence of a single or a cluster of RL. Specifically, it was shown that for implanted transducers and 0.5 MHz, as well as for both the hypothetic case of homogeneous and the realistic of nonhomogeneous bone, the presence of one or more RLs can be identified by the receivers which are placed directly above the region of interest revealing the effectiveness of the FAS wave as an indicator. The same observation was made for all the sizes, numbers, depths and lengths of the RL. However, the use of 1 MHz or the transducers’ placement into the water led to the detection of larger pores with a short delay when the RLs are placed in the center of the plate (approximately 4 mm) or near the upper cortical cortex (approximately 2 mm). It should be emphasized that the occurrence of a single RL near the lower surface of cortical bone could not be identified when the excitation frequency 1 MHz was applied irrespective of the transducers’ configuration. In addition, it was observed that as the receivers are removed from the region of interest the calculated values gradually approximate the values of healthy bone. Nonetheless, the combination of low and high frequencies for the detection of larger pores could be useful, as low frequencies can detect the region of interest along the *x* axis and higher frequencies can reveal the depth of the pores along the *y*-axis. For example, in Figure 9b,e, when the RL is placed near the upper cortical layer (grey line), for both frequencies, we can detect the large pore in the same position implying that the pore is near the cortical upper surface. If we place the RL in the center of the plate (red line), the use of 0.5 MHz frequency leads to the detection of the exact *x* position of the RL, but 1 MHz shows a delay. This reveals that the pore is placed deeper. Finally, Figure 9e shows that for 1 MHz we cannot detect the RL at *y* = 3 (purple line), indicating in combination with 0.5 MHz findings that a large pore exist, but this pore is positioned near the lower surface of the cortical cortex. Thus, even from the limitations of 1 MHz, we may derive specific information concerning the depth of the RL interpreted in combination with 0.5 MHz findings when the occurrence of large basic multicellular units is a factor of major concern.

A possible explanation for the low sensitivity of 1 MHz is that, when the wavelengths are comparable to or smaller (*λ_0.5MHz_* = 7.88 mm, *λ_1MHz_* = 3.94 mm), the thickness of the cortical cortex (4 mm), the FAS wave propagates as a subsurface, lateral wave which travels at the bulk longitudinal velocity of the medium and cannot reflect changes occurring at deeper cortical layers. The calculation of the wavelenths depends on the frequency and the bone’s bulk velocity which is 3939 m/s considering the material properties of Table 1. However, bone velocity may vary from 3500 m/s to 4100 m/s depending on the type and anatomic region of bone as well as cortical thickness. According to the literature [15,28], the use of low excitation frequencies (0.2 MHz) has been chosen to yield information on the correlation of the FAS velocity with changes in cortical thickness due to osteoporosis, while it was reported in [28] that the FAS velocities for high frequencies such as 1 MHz were weakly correlated with cortical thicknesses. Therefore, future research should address the question of which frequency range below 0.5 MHz is more convenient for the assessment of osteoporosis considering the occurrence of pores with different sizes.

Concerning the case of homogeneous bone, it was observed that, for 1 MHz, the FAS velocity along the propagation path increases slightly, approximating the bone’s bulk velocity (Figure 9d–f), while this is not the case for 0.5 MHz in Figure 9a–c. The observed behavior at 0.5 MHz can be attributed to the fact that the wavelength is almost twice the cortical thickness implying that more complex wave propagation phenomena evolve and the investigation of the propagation of guided waves could convey significant information. On the other hand, for 1 MHz, the FAS propagates as a lateral wave.

Finally, several assumptions were made which require further numerical research and improvements. First of all, although two dimensional geometries were developed, they incorporated various porosity scenarios. Moreover, changes in cortical thickness in cases of pathology in combination with changes in cortical porosity were not considered. Imaging modalities could be also exploited to establish more realistic computational models [29,30]. In addition, the use of point transducers should be mentioned as in experimental procedures the transducers have a finite size. It was reported in [4] that the choice of using point elements instead of finite-sized ones did not affect significantly the recorded ultrasound velocities. According to our previous study using numerical models from SAM images [31], the application of transducers with a finite size larger than the RL diameter could provide significant information concerning the occurrence of a region of RLs. However, further numerical research is needed to investigate the impact of the transducers’ size in relation to the scatterers’ size on the results. Furthermore, according to [32], the majority of patients with osteoporosis have changes in the mineralization pattern. In particular, reduction of average mineral content was described when compared with agematched controls or when compared with reference values for healthy adults. Quantitative ultrasound was used in [33] for bone characterization and the role of bone mineral density as a major determinant of acoustic properties was highlighted as well as the significance of density-independent relationships with bone microarchitecture. However, according to [34], the main cause of sound attenuation coefficient in bone is porosity, while matrix stiffness has a minor effect. In addition, it was reported in [19] that in aged women the changes in porosity prevail over those of of matrix elasticity to drive the variations of the bone mesoscopic elasticity.

Nevertheless, the results clearly indicated that the excitation frequency 0.5 MHz is more sensitive to detect changes in cortical porosity and the occurrence of RLs. In addition, it was shown that the calculation of the FAS velocity in small propagation paths could potentially provide significant quantitative information for the early diagnosis of osteoporosis and detection of pathologic regions with larger pores with a high risk for fracture.

## 6. Conclusions

In this work, we presented a systematic and parametric computational study on ultrasound wave propagation in healthy and osteoporotic bones. The FAS velocity was derived in small propagation paths to extract quantitative criteria for the diagnosis and assessment of osteoporosis taking into consideration not only total changes in cortical porosity, but also the occurrence of larger pores and various microstructural features. The role of the excitation central frequency and the impact of the transducers’ positions on the FAS velocity variation profile were examined. For the first time, the gradual formation of osteoporosis was also considered. It was shown that the local FAS velocities derived from a proper combination of low and high frequencies may contribute to the prevention or monitoring of osteoporosis and reveal potential pathologic regions with larger pores at an early stage of the disease. Further numerical research is suggested on challenges for clinical application and more realistic scenarios using scanning acoustic microscopy images.

## Figures and Tables

**Table 1:** Elastic coefficients and density used for the simulations [14].

Propagation Medium	C_11_ (GPa)	C_12_ (GPa)	C_66_ (GPa)	*Ρ* (g/cm^3^)
Water	2.25	2.25	0	1.00
Bone	28.71	10.67	9.02	1.85

**Table 2:** Examined cases in the tangential direction for the second set of the simulations.

	Description	Porosity (%)	No. of Pores	Pores’ Radius (μm)	No. of RL/RLs	Radius of RL/RLs (μm)
B_Hom	Homogeneous bone	0	0	–	–	–
B_Po5	Porous bone, normal pores	5	1592	40	–	–
B_Po5_RL1	Porous bone, normal pores and 1 RL	5	1592	40	1	115
B_Po5_RL3	Porous bone, normal pores and 3 RLs	5	1592	40	3	115
B_Po5_RL5	Porous bone, normal pores and 5 RLs	5	1592	40	5	115
B_Po10	Porous bone, normal pores	10	1592	60	–	–
B_Po16	Porous bone, normal pores	16	2263	60	–	–
B_Po16_RL	Porous bone, normal pores and RLs	16	1400	60	235	115
B_Po16_Gradual	Gradual distribution of the pores, normal pores and RLs	16	1775	40, 60, 80	192	115

**Table 3:** Linear fit of the mean first arriving signal (FAS) velocities derived from all the receiving positions and the excitation frequency 0.5 MHz.

	Implanted Transducers			Transducers at a Distance of 2 mm from the Cortical Surface		
	*y* = a*x* + b	R^2^	RMSE	*y* = a*x* + b	R^2^	RMSE
B_Hom	*y* = 2.27*x* + 3844	0.54	16.56	*y* = 3.00*x* + 3815	0.52	22.79
B_Po5	*y* = 0.17*x* + 3661	0.01	37.94	*y* = 2.81*x* + 3577	0.17	48.84
B_Po10	*y* = 0.72*x* + 3475	0.01	78.51	*y* = 0.28*x* + 3489	0.01	66.92
B_Po16	*y* = −4.37*x* + 3417	0.09	110.08	*y* = −3.64*x* + 3385	0.53	24.58
B_Po16_RL	*y* = −4.09*x* + 3434	0.06	123.45	*y* = −3.23*x* + 3410	0.06	92.93
B_Po16_Gradual	*y* = 1.38*x* + 3409	0.02	75.88	*y* = 1.57*x* + 3397	0.03	59.72

**Table 4:** Linear fit of the mean FAS velocities derived from all the receiving positions and the excitation frequency 1 MHz.

	Implanted Transducers			Transducers at a Distance of 2 mm from the Cortical Surface		
	*y* = a*x* + b	R^2^	RMSE	*y* = a*x* + b	R^2^	RMSE
B_Hom	*y* = 1.08*x* + 3898	0.80	4.26	*y* = 1.65*x* + 3879	0.80	6.37
B_Po5	*y* = −1.41*x* + 3748	0.28	17.82	*y* = −1.81*x* + 3759	0.19	29.16
B_Po10	*y* = −1.54*x* + 3639	0.02	93.23	*y* = −1.78*x* + 3628	0.06	57.31
B_Po16	*y* = −1.36*x* + 3549	0.01	100.58	*y* = −0.06*x* + 3510	0.01	30.84
B_Po16_RL	*y* = −5.38*x* +3 702	0.13	111.10	*y* = −3.21*x* + 3647	0.28	36.73
B_Po16_Gradual	*y* = 3.96*x* + 3440	0.15	72.96	*y* = 4.49*x* + 3406	0.16	73.61
